# Predicted networks of protein-protein interactions in *Stegodyphus mimosarum* by cross-species comparisons

**DOI:** 10.1186/s12864-017-4085-8

**Published:** 2017-09-11

**Authors:** Xiu Wang, Yongfeng Jin

**Affiliations:** 1 Institute of Ecology, College of Life Sciences, Zhejiang University, Hangzhou Zhejiang, ZJ310058 People’s Republic of China; 2 Institute of Biochemistry, College of Life Sciences, Zhejiang University, Hangzhou Zhejiang, ZJ310058 People’s Republic of China

**Keywords:** *Stegodyphus mimosarum*, Protein–protein interaction, Orthologue, Protein network

## Abstract

**Background:**

*Stegodyphus mimosarum* is a candidate model organism belonging to the class Arachnida in the phylum Arthropoda. Studies on the biology of *S. mimosarum* over the past several decades have consisted of behavioral research and comparison of gene sequences based on the assembled genome sequence. Given the lack of systematic protein analyses and the rich source of information in the genome, we predicted the relationships of proteins in *S. mimosarum* by bioinformatics comparison with genome-wide proteins from select model organisms using gene mapping.

**Results:**

The protein–protein interactions (PPIs) of 11 organisms were integrated from four databases (BioGrid, InAct, MINT, and DIP). Here, we present comprehensive prediction and analysis of 3810 proteins in *S. mimosarum* with regard to interactions between proteins using PPI data of organisms. Interestingly, a portion of the protein interactions conserved among *Saccharomyces cerevisiae*, *Homo sapiens*, *Arabidopsis thaliana*, and *Drosophila melanogaster* were found to be associated with RNA splicing. In addition, overlap of predicted PPIs in reference organisms, Gene Ontology, and topology models in *S. mimosarum* are also reported.

**Conclusions:**

Addition of *Stegodyphus,* a spider representative of interactomic research, provides the possibility of obtaining deeper insights into the evolution of PPI networks among different animal species. This work largely supports the utility of the “stratus clouds” model for predicted PPIs, providing a roadmap for integrative systems biology in *S. mimosarum*.

**Electronic supplementary material:**

The online version of this article doi:(10.1186/s12864-017-4085-8) contains supplementary material, which is available to authorized users.

## Significance

*S. mimosarum* is a candidate model organism in which to study biology and evolution. Analysis of protein–protein interaction (PPI) networks, especially the whole PPI network in a given species, provides useful information regarding protein function and signaling pathways. In addition, analysis of the whole and partial PPI networks from the perspective of topology is beneficial for understanding the functions of protein nodes. However, little information is available regarding the whole PPI network in *S. mimosarum*. Therefore, we constructed and analyzed the whole PPI network in *S. mimosarum* by gene mapping. This represents the first attempt to analyse PPI sub-networks implicated in RNA splicing with reference to the study of protein function associated with the RNA splicing process from a new perspective. Gene mapping is computationally inexpensive, and was chosen above other algorithms due to the rapidity of the analysis and the low error rate.

## Background

Protein–protein interactions (PPIs) are involved in most of the activities of life [1]. Knowledge of PPI networks will facilitate molecular studies on diverse biological processes and insight into the biology of proteins with no known function in a specific species. Over the past several decades, experimental methods have been developed to study PPIs, such as yeast two-hybrid screening [2, 3], affinity chromatography [4], co-precipitation [5], fluorescence resonance energy transfer [6], protein chip [7], and the yeast three-hybrid system [8]. The increasing number of experimentally determined protein interactions has made it possible to systematically identify PPIs. However, experimental PPI data for construction of whole PPI networks in a given species are still limited. Since the turn of the millennium, high-throughput computational approaches, such as phylogenetic profiling [9], gene neighbour [10], and interologue [11], have been developed to investigate protein interaction relationships on a proteome-wide scale.

Experimental mapping of large-scale protein–protein interaction networks has been performed in several species. The first complete PPI map was obtained for *Saccharomyces cerevisiae* [12], followed by other organisms, including *Caenorhabditis elegans*, *Drosophila melanogaster*, *Homo sapiens*, *Arabidopsis thaliana*, *Oryza sativa*, and *Coffea arabica* [13–18]. In addition to *A. thaliana*, the PPI map of the plant species *Physcomitrella patens* was also reported [19]. Due to the large volume of both quantitative and qualitative PPI data, several PPI databases have been generated, such as BioGrid, MINT, BIND, and DIP, which provide useful resources for inferring the biological significance underlying PPI networks for both model and non-model organisms. Unfortunately, data on PPIs in model organisms with distant evolutionary relationships are limited in the public databases.

The interologue method, which is based on the evolutionary scenario that if proteins are conserved among different species, then the interactions between the two proteins in one organism are also likely to exist in these different species, has become a useful bioinformatics approach for drawing PPI maps. To predict interologues by conservation, it is necessary to obtain an accurate set of orthologues. The BLAST sequence alignment algorithm is used for identification of orthologues between species [20]. Such cross-species mapping has facilitated the development of websites related to orthologues in various species, such as STRING and InParanoid [21–23].

Spiders attract wide interest because of their biochemical and structural properties, pharmacological and pathophysiological systems, and evolutionary significance [24–27]. *Stegodyphus mimosarum* is one of very few cooperatives spiders. Based on a partial molecular phylogeny of the genus *Stegodyphus*, the hypothesis of spider socialilty (i.e., that social spiders in this genus are evolutionary transient) has been addressed [28, 29]. Phylogenetic analysis of the genus *Stegodyphus* suggests that sociality is associated with reduced effectiveness of selection [29]. Additionally, dispersal by ballooning also appears to have been observed early in *Stegodyphus mimosarum* [30], and has been shown to exist in other *Stegodyphus* [31]. Assembled genome sequences, transcriptome sequences, and orthologous genes in *S. mimosarum* have provided new opportunities to gain insight into these properties [32, 33].

Although some knowledge about the phylogeny and evolution of *S. mimosarum* is available, there have been few systematic analyses of protein–protein interactions in *Stegodyphus* species. Moreover, compared with well-established protein–protein interactions in *Drosophila*, the information about PPI networks is little known in Chelicerata, the second largest group of terrestrial animals. Here, we propose a computational method that can be used to predict PPIs in *S. mimosarum* using publicly available protein sequence databases. This provides an outline of conserved eukaryotic biological pathways, which will aid in current research and provide a framework for future interactomics research in Arthropoda.

## Methods

### Interactome data collection of organisms

Interactome data were collected from public PPI databases such as BioGrid, InAct, DIP, BIND, and SGD (only for PPI data in *S. cerevisiae*). Eleven organisms were used for PPI network construction because of the rich resources available: *S. cerevisiae*, *C. elegans*, *D. melanogaster*, *H. sapiens*, *E. coli*, *A. thaliana*, *M. musculus*, *D. rerio*, *R. norvegicus*, *P*. *falciparum*, and *C*. *jejuni*.

### Construction of interactome database from organisms

PPIs were downloaded from public databases, and standard identifiers developed for each interactor. Universal reference interactomes from these public databases have several identifier IDs, UniProt ID, Ensembl ID, Entrez ID, and RefSeq ID. UniProt ID was selected as the standard format for ID exchange of other identifiers due to the maturity of the UniProt website. The data were output in standard format and imported into a local MySQL database. Standard PPI data were divided into 11 groups representing the 11 chosen organisms. Our local SQL database contained each unique combination of interactor A and B, including proteins that interact with A–B. Multiple A–B or B–A entries were counted as a single interaction, and were integrated to remove redundancy.

### Orthologue prediction

Genome-wide protein sequences in organisms, namely *C. elegans*, *D. melanogaster*, *H. sapiens*, *E. coli*, *A. thaliana*, *M. musculus*, *D. rerio*, *R. norvegicus*, *P*. *falciparum*, *C*. *jejuni*, and *S. cerevisiae*, as well as *S. mimosarum*, were retrieved from the NCBI website. To obtain as many functional orthologues as possible in these organisms, we used InParanoid 4.1 software to separated orthologues and outparalogues after comparison between organisms and *S. mimosarum*, respectively. Where multiple orthologue groups were possible, such as one-to-many and many-to-many orthologues, only a one-to-one orthology was created. To avoid generating too much predicted data and thus reducing the accuracy of the prediction results, we used orthologue score 1.0 data among conserved proteins. The chosen orthologs in organisms to *S. mimosarum* were entered into the MySQL local database.

### Construction of *S. mimosarum* interactome

**Fig. 1:**
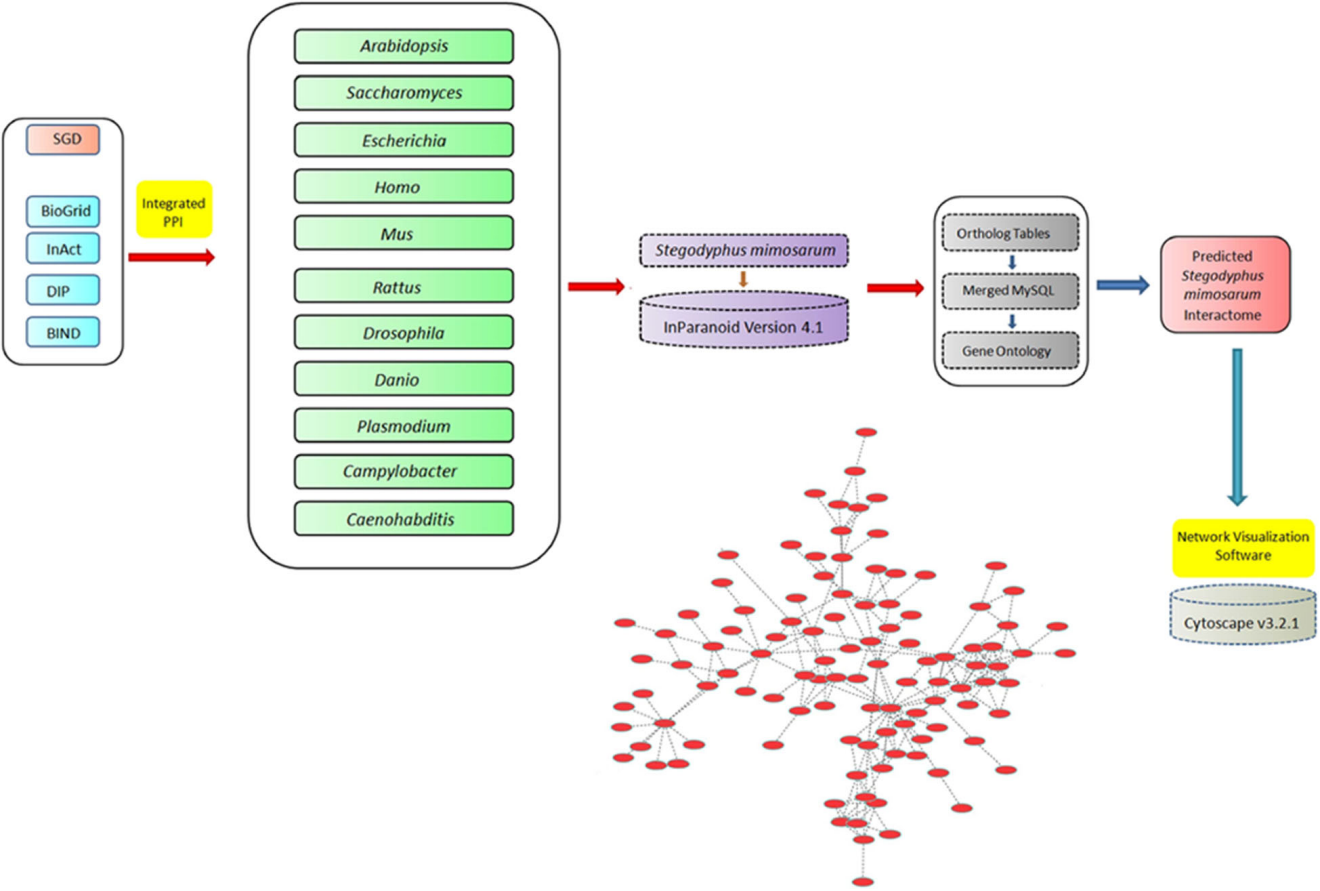
Flowchart for the predicted *S. mimosarum* interactome. The predicted interactome in *S. mimosarum* was derived from orthologs of 11 organisms using InParanoid 4.1. The PPI dataset was used to query a MySQL database containing PPIs from SGD, BioGrid, InAct, DIP, and BIND databases. The predicted *S. mimosarum* interactome and supporting information were input into Cytoscape v3.2.1 for visualization

The chosen one-to-one orthologs from organisms to *S. mimosarum* were used to match proteins in *S. mimosarum* according to corresponding relationships of PPI in organisms, which were also loaded into the local MySQL database. The local database contained all data for predicted PPI in *S. mimosarum*, and included the unique *S. mimosarum* interactome, organisms, predicted PPI data, and UniProt ID for reference interactions. To visualize the PPI network in *S. mimosarum*, the predicted PPI data in *S. mimosarum* were loaded into Cytoscape v3.2.1. The general process for assembling the database is outlined in the flow chart shown in Fig. 1.

### Gene ontology analysis of PPI data

GO annotations based on the predicted proteins in the PPI network of *S. mimosarum* were downloaded from the GO database and the UniProt website. First, GenBank IDs in *S. mimosarum* selected corresponding to protein IDs in Retrieve/ID mapping at the UniProt website were used to obtain Uniprot IDs and GO annotation mapping using the “columns” option. Based on GO classifications, some of the genes were mapped to “biological processes”, and some to “molecular function”. Second, GO annotation data from *S. mimosarum* were used with the download option to get additional files. Gene Ontology IDs for “over-representation” were calculated using Stata software. Highly connected hubs from the constructed PPI network in *S. mimosarum* and model organisms were also annotated according to the database and UniProt website.

## Results

### PPI datasets of organisms

**Fig. 2:**
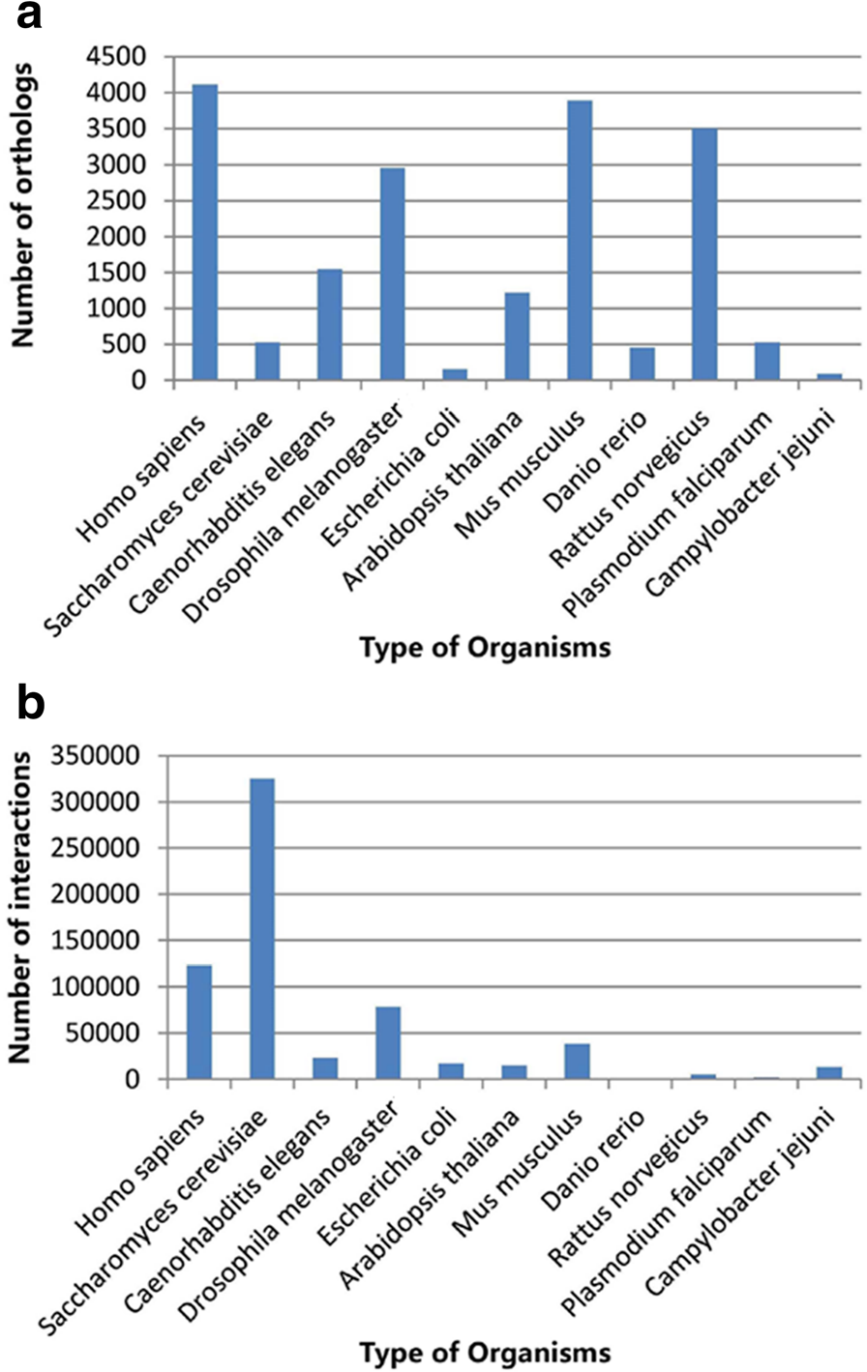
The numbers of *S. mimosarum* gene orthologues and interactions in model organisms. **a** Histogram showing number of interactions by model organism. **b** Histogram of orthologues by model organism

Interactions of proteins in one organism are expected to be conserved in other related organisms. An interspecies comparison of PPI data from 11 organisms was recently carried out to identify conserved networks. These datasets from the organisms could be used to inspect the quality of PPI predictions in *S. mimosarum*. Overall, 123,650 interactions in *H. sapiens*, 325,102 in *S. cerevisiae*, 23,241 in *C. elegans*, 78,525 in *D. melanogaster*, 17,428 in *E. coli*, 15,195 in *A. thaliana*, 38,719 in *M. musculus*, 449 in *D. rerio*, 5096 in *R. norvegicus*, 2248 in *P*. *falciparum*, and 13,676 in *C*. *jejuni* for which data were available were collected from five PPI databases (see Materials and Methods). As indicated by these organism datasets, differences in the number of PPIs could be explained by the different amounts of experimental data for the organisms, along with which major experimental methods, including yeast two-hybrid, affinity chromatography, co-precipitation, fluorescence resonance energy transfer, and protein chip, were used. These analyses confirmed that the greatest number of quality PPIs was found in *S. cerevisiae* and the lowest number was found in *D. rerio*, corresponding to the largest and smallest experimental PPI data set in recent years, respectively (Fig. 2b). In addition, the number of high-quality interactions in mammals, especially in *H. sapiens*, could be due to gene duplication in these organisms, which likely led to multifunctionalisation and sub-functionalisation because of selection pressure. Given the large numbers of isoforms, PPI numbers from mammals would be higher than those in other organisms.

### Prediction of *S. mimosarum* orthologues

To identify orthologues, proteome-wide sequence comparisons between 11 organisms and *S. mimosarum* were performed using InParanoid 4.1 (see Materials and Methods), which is based on BLAST search, followed by orthologous group clustering. A total of 27,135 proteins were annotated from the whole genome of *S. mimosarum*. The numbers of orthologous protein pairs between *S. mimosarum* and each of the organisms are shown in Fig. 2a. Briefly, 4112 *H. sapiens*, 3888 *M. musculus*, 3506 *R. norvegicus*, 2959 *D. melanogaster*, 1550 *C. elegans*, 1224 *A. thaliana*, 532 *P*. *falciparum*, 529 *S. cerevisiae*, 456 *D. rerio*, 91 *E. coli*, and 155 *C*. *jejuni* gene groups had high-confidence orthologues (1.0) in *S. mimosarum*, and were regarded as the most highly conserved genes among these organisms and *S. mimosarum*. *H. sapiens* and *D. rerio* showed the greatest and least number of proteins in orthologous groups with *S. mimosarum*, respectively. The number of PPIs was not related to closeness of genetic relationship, as indicated by the greatest number orthologues in the comparison between *S. mimosarum* and *H. sapiens*, likely due to the large amount of genome information available for *H. sapiens*.

To confirm conserved orthologues between model organisms and *S. mimosarum*, overlapping orthologues in *S. mimosarum* were chosen for analysis in different groups based on species with closer and more distant relationships. A total of 3495 orthologues in *S. mimosarum* were common to the datasets of *H. sapiens* and *M. musculus*, and eight orthologues in *S. mimosarum* were common to the datasets of *D. rerio* and *C*. *jejuni*. The former relatively rich overlap was not surprising given the large number of orthologues in *H. sapiens* and *M. musculus*. The latter relative lack of overlap was also not surprising given the low number of orthologues in *D. rerio* and *C*. *jejuni*. Substantial numbers of *S. mimosarum* genes exhibited orthologues in two species (Table 1). Species with closer relationships were suggested to show larger degrees of overlap, whereas less overlap was observed with those that had more distant relationships.

**Table 1:** Overlap of *S. mimosarum* orthologs between two genomes

	*Homo*	*Mus*	*Rattus*	*Danio*	*Drosophila*	*Caenohabditis*	*Arabidopsis*	*Escherichia*	*Saccharomyces*	*Campylobacter*	*Plasmodium*
*Homo*		3495	3279	378	2318	1299	1077	119	494	66	492
*Mus*			3340	379	2298	1296	1068	120	491	65	494
*Rattus*	3270	3340		369	2262	1285	1046	116	494	63	495
*Danio*	378	379	369		331	213	183	9	104	8	106
*Drosophila*	2318	2298	2262	331		1253	997	113	493	64	482
*Caenohabditis*	1299	1296	1285	213	1253		727	92	415	52	414
*Arabidopsis*	1077	1068	1046	183	997	727		85	413	44	448
*Escherichia*	119	120	116	9	113	92	85		64	53	40
*Saccharomyces*										35	296

The Overlap were obtained from ortholog in two model organism to *S. mimosarum*, respectively. Each model organism represents orthologs in each model organism to *S. mimosarum*

The orthologue pairs between model organisms and *S. mimosarum* were also used to systematically examine the overlap among more than two species. At maximum, 3178 orthologs in *S. mimosarum* were conserved among *H. sapiens*, *M. musculus*, and *R. norvegicus*. At minimum, eight orthologs in *S. mimosarum* were conserved among *H. sapiens*, *M. musculus*, *R. norveicus*, *D. rerio*, *D. melanogaster*, *C. elegans*, and *E. coli* (Table 2). The results of statistical analyses also suggested that these highly conserved orthologues are likely to have important protein functions in *S. mimosarum*. Therefore, these conserved orthologue pairs were mainly used to systematically examine the overlap among the predicted protein interactions in *S. mimosarum*.

**Table 2:** Overlaps of *S. mimosarum* orthologs across organism genomes

	*Homo*	*Mus*	*Rattus*	*Danio*	*Drosophila*	*Caenohabditis*	*Arabidopsis*	*Escherichia*	*Saccharomyces*	*Campylobacter*	*Plasmodium*
3178	+	+	+								
344	+	+	+	+							
290	+	+	+	+	+						
182	+	+	+	+	+	+					
133	+	+	+	+	+	+	+				
6	+	+	+	+	+	+	+	+			
8	+	+	+	+	+	+		+			
93	+	+	+	+	+	+			+		
8	+	+	+	+	+	+				+	
91	+	+	+	+	+	+					+

The ortholog in each model organism to *S. mimosarum* were used for obtaining overlaps. “**+**” represents the ortholog in each model organism to *S. mimosarum*

To determine whether there are overlaps in other *Stegodyphus* species related to the overlaps among evolutionarily distant model organisms and *S. mimosarum*, we first searched for conserved orthologs in *Stegodyphus* species, including *S. lineatus*, *S. tentoriicola*, and *S. mimosarum*. A total of 1184 GenBank IDs in *S. mimosarum* with overlapping orthologs in all three social *Stegodyphus* species were obtained from the NCBI website [32]. Three proteins, KFM80602.1, KFM79040.1, and KFM69424.1, were found to overlap between the orthologues of *Stegodyphus* species and those of the evolutionarily distant organisms *C. elegans*, *D. melanogaster*, *H. sapiens*, *M. musculus*, *R. norvegicus*, *D. rerio*, *C*. *jejuni*, *S. cerevisiae*, *E. coli*, and *A. thaliana*. Histidine triad nucleotide-binding protein 1 (KFM80602.1), GTP-binding protein (KFM79040.1), and heat shock protein (KFM69424.1) were annotated as having catalytic activity, ATP binding function, and GTP-binding function, respectively.

### Analysis of protein–protein interactions of *S. mimosarum*

Based on the PPI datasets of the organisms in the MySQL database, a total of 58,489 protein–protein interactions were predicted from 3810 different proteins of *S. mimosarum* using the PPI datasets of 11 organisms (Additional file 1). The majority of predicted PPIs from *S. mimosarum* were provided by comparison of PPIs from *H. sapiens*, *D. melanogaster*, and *S. cerevisiae*. Specifically, 38% of all data came directly from experiments in *H. sapiens*, 23% from *D. melanogaster*, and 25% from *S. cerevisiae*. However, there were only a few predicted PPIs from *D. rerio* and *P*. *falciparum*. Taken together, these numbers from different model organisms indicated possible overlaps could be quantified. In addition, the data of orthologous pairs were consistent with the corresponding amounts of PPI data for each species; 38% of interactions matched with human, whereas only 9% corresponded to those in mice. Given the different PPI resources in human and mouse from public databases, inconsistency between the number of orthologs and PPIs may be reasonable.

**Fig. 3:**
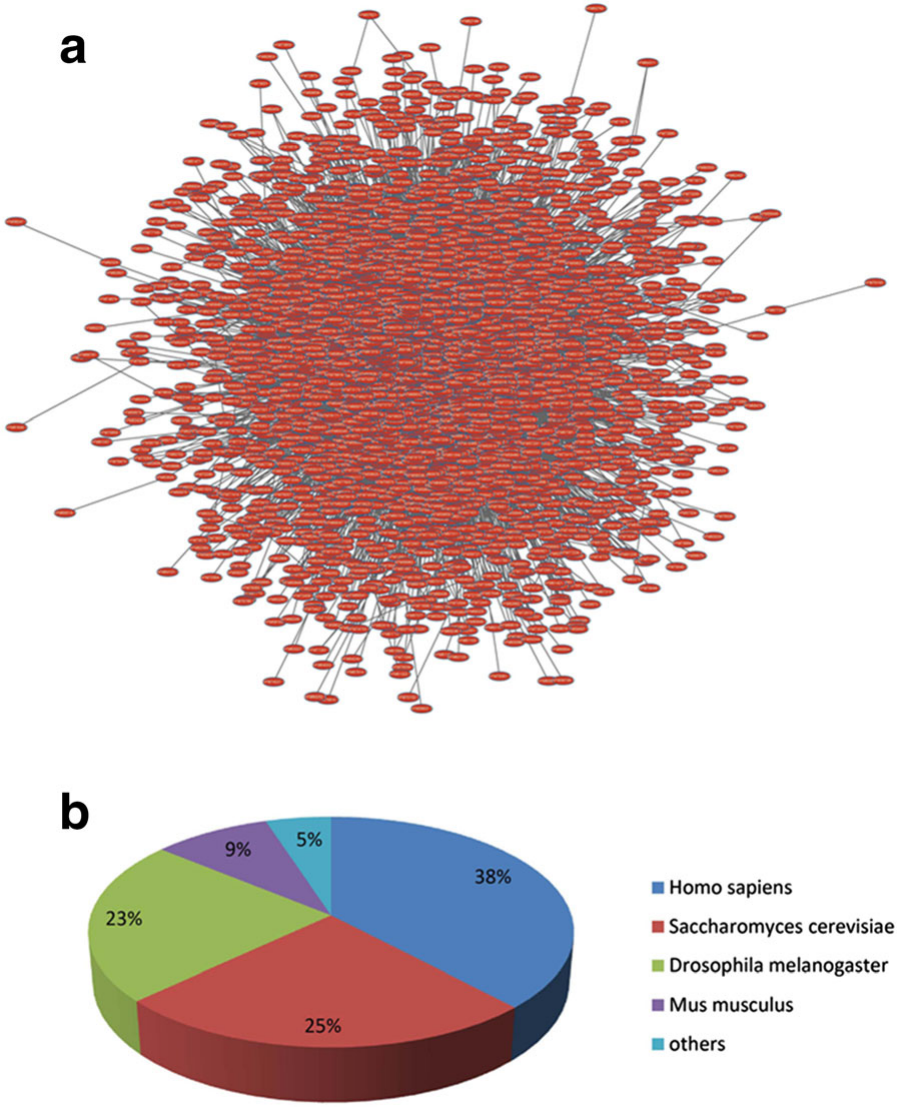
The *S. mimosarum* network was viewed in Cytoscape v3.2.1, and the predicted interactions distribution of *S. mimosarum* were analysed in the model organisms. **a** Large ball of 58,489 non-redundant interactions in the normal view of Cytoscape v3.2.1. **b** The predicted interaction distribution of *S. mimosarum* by model organism; *H. sapiens* provided the largest number of interactions for *S. mimosarum* PPI prediction, followed by *S. cerevisiae* and *M. musculus*

**Fig. 4:**
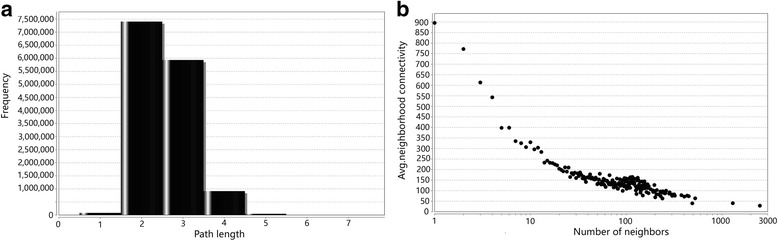
Analysis of *S. mimosarum* interaction network with 3810 nodes in Cytoscape v3.2.1. **a** The edge frequency in different path lengths. Path length means edge length. This indicates that the edge frequency is dominated by short path length (1–3). **b** The relation between neighbourhood connectivity and number. The decreasing trend of the neighborhood connectivity shows high clustering coefficient in neighbor nodes of relative lower connectivity

**Fig. 5:**
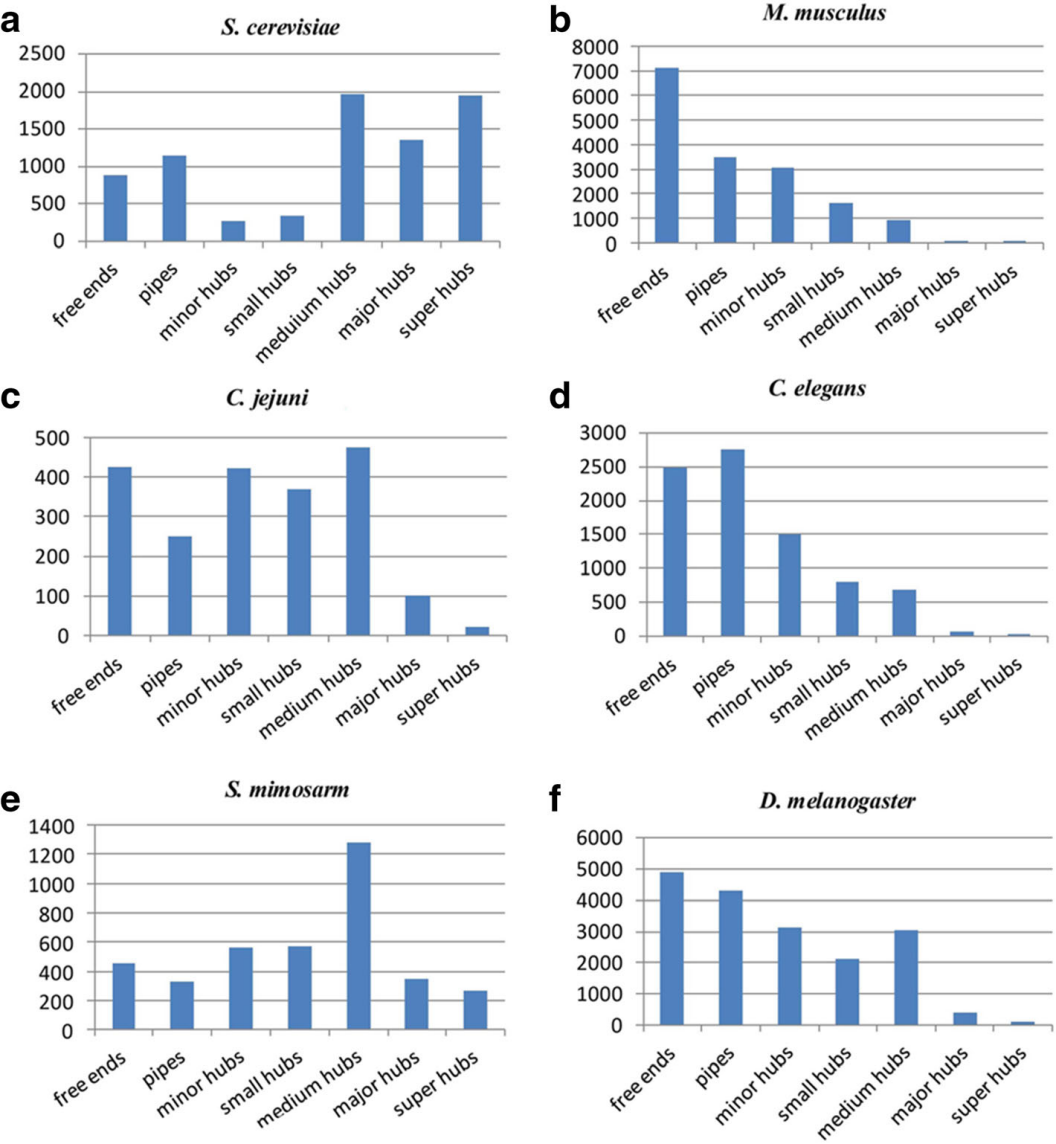
Distribution of hub types among *S. cerevisiae*, *C. elegans*, *D. melanogaster*, *M. musculus*, *C*. *jejuni*, and *S. mimosarum*. **a** *S. cerevisiae* (experimentally determined; from BioGrid, InAct, MINT, DIP, and SGD). **b** *M. musculus* (experimentally determined; from BioGrid, InAct, MINT, DIP). **c** *C*. *jejuni* (experimentally determined; from BioGrid, InAct, MINT, and DIP). **d** *C. elegans* (experimentally determined; from BioGrid, InAct, MINT, and DIP). **e** *S. mimosarum* (predicted, this work). **f** *D. melanogaster* (experimentally determined; from BioGrid, InAct, MINT, and DIP)

After predicting PPIs in *S. mimosarum*, the PPI network was visualized using Cytoscape v3.2.1. The “normal layout” was used for the predicted PPI network, and the “network analyser” tool was used for PPI network analysis (Fig. 3). The PPI network was calculated to show the topological parameters, represented as single values and distributions (Table 3). Analysis of interaction network showed the short path length distribution and the decreasing trend of neighbourhood connectivity distribution (Fig. 4a and b). This indicated that the network possessed small-world property as previously reported in human protein interaction network for neurodegenerative diseases [34], suggesting the reliability of this prediction*.* To analyse the hub types of the network, several types of hub were computed in *S. mimosarum*, including free ends (only one interaction), pipes (two interactions), minor hubs (3–5 interactions), small hubs (6–11 interactions), medium-sized hubs (12–50 interactions), and other hubs of different sizes. A total of 3810 protein hubs were evaluated by the whole-network topology based on the interaction numbers with *S. mimosarum*. Specifically, the most common hub type in *S. mimosarum* consisted of medium hubs ranging in size from 12 to 50 interactions. In addition to *S. mimosarum*, medium-sized hubs were also detected in *S. cerevisiae*, *C*. *jejuni*, and *P. patens* [19]. When major and medium-sized hubs were primary hubs in organisms, minor hubs, pipes, and free ends could easily be under-represented. The largest numbers of free ends were detected in the networks of the two model species *D. melanogaster* and *M. musculus*, whereas pipes were most abundantly detected in *C. elegans* (Fig. 5).

**Table 3:** Analysis of the interaction network topology of *S. mimosarum*

Topology Parameters	Score
Clustering coefficiet	0.286
Connected components	12
Network diameter	7
Network radius	1
Network centralization	0.647
Shortest paths	99%
Characteristic path length	2.544
Number of neighbors	22.214
Number of nodes	3810
Network density	0.006
Network heterogeneity	2.640
Lsolated nodes	8
Multi-edge node pair	15,265

### Analysis of highly connected nodes

**Fig. 6:**
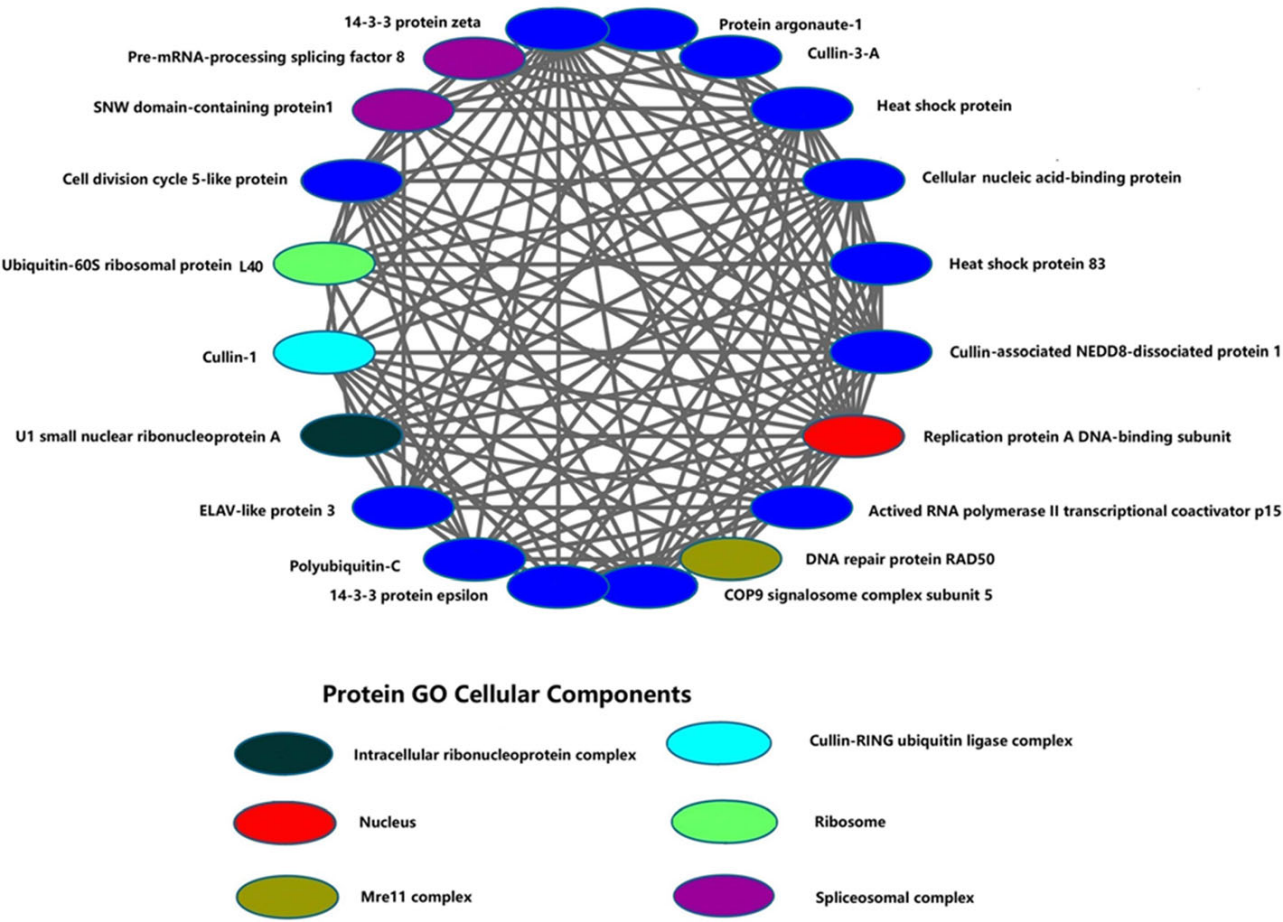
The top 20 hubs in the predicted PPI of *S. mimosarum* were elucidated from the network. Twenty hubs represent the most highly connected proteins in the predicted PPI of *S. mimosarum*

Normally, essential genes are highly interconnected hubs at the protein level. The search for highly connected hubs indicated that many of the interacting proteins in these hubs were functionally related to mRNA splicing, protein folding, DNA repair, cell division, regulation of transcription, or ubiquitin-dependent protein catabolic processes (Fig. 6). The top 20 highly connected protein hubs were identified from *S. mimosarum*. The maximum number of nodes in the top 20 proteins was 2768, and the minimum number was 359 (Additional file 2). Polyubiquitin-C protein (KFM70679.1) was identified as the node most frequently present within the large hubs of the PPI network in *S. mimosarum*. The possible explanation for its high degree of connection is that ubiquitin is involved in a variety of biological processes, including neural development, spermatogenesis, egg production, and fertilisation. Despite its being the largest hub in *S. mimosarum*, it is surprising that polyubiquitin-C protein was not the node with the highest degree of connectivity in the other model organisms. This may have been because the type of node model may not be the same in other model organisms, and the amounts of protein PPI information were also limited in some organisms [35]. The proteins 14–3-3 protein epsilon (KFM63563.1), 14–3-3 protein zeta (KFM75839.1), and pre-mRNA-processing-splicing factor 8 (KFM66933.1) were identified among the highly connected proteins in *M. musculus*, *D. melanogaster*, and *H. sapiens*, respectively [36]. Similar to heat shock proteins (HSP 90 and HSP 60), which were among the highly connected proteins in *P. patens*, heat shock protein (HSP83) (KFM78806.1) was also identified among the top 20 node proteins in *S. mimosarum* [37]. This protein was shown to have many orthologs in the organisms *D. melanogaster*, *M. musculus*, *A. thaliana*, *S. cerevisiae*, *C. elegans*, *H. sapiens*, *E. coli*, and *C*. *jejuni*. This may be because HSP83 is involved in highly conserved pathways and functions in these species. It is worth noting that pre-mRNA-processing-splicing factor 8 (KFM66933.1) and SNW domain-containing protein 1 (KFM68418.1), two genes associated with the mRNA splicing process, were among the most highly connected nodes in *S. mimosarum*.

Pre-mRNA-processing-splicing factor 8 functions in U5 or U6 snRNA binding; the function of SNW domain-containing protein 1 is less well understood. Cullin-3-A protein, which is involved in ubiquitin-dependent protein catabolism, is also an interesting node (Fig. 6).

### Gene ontology analysis

**Fig. 7:**
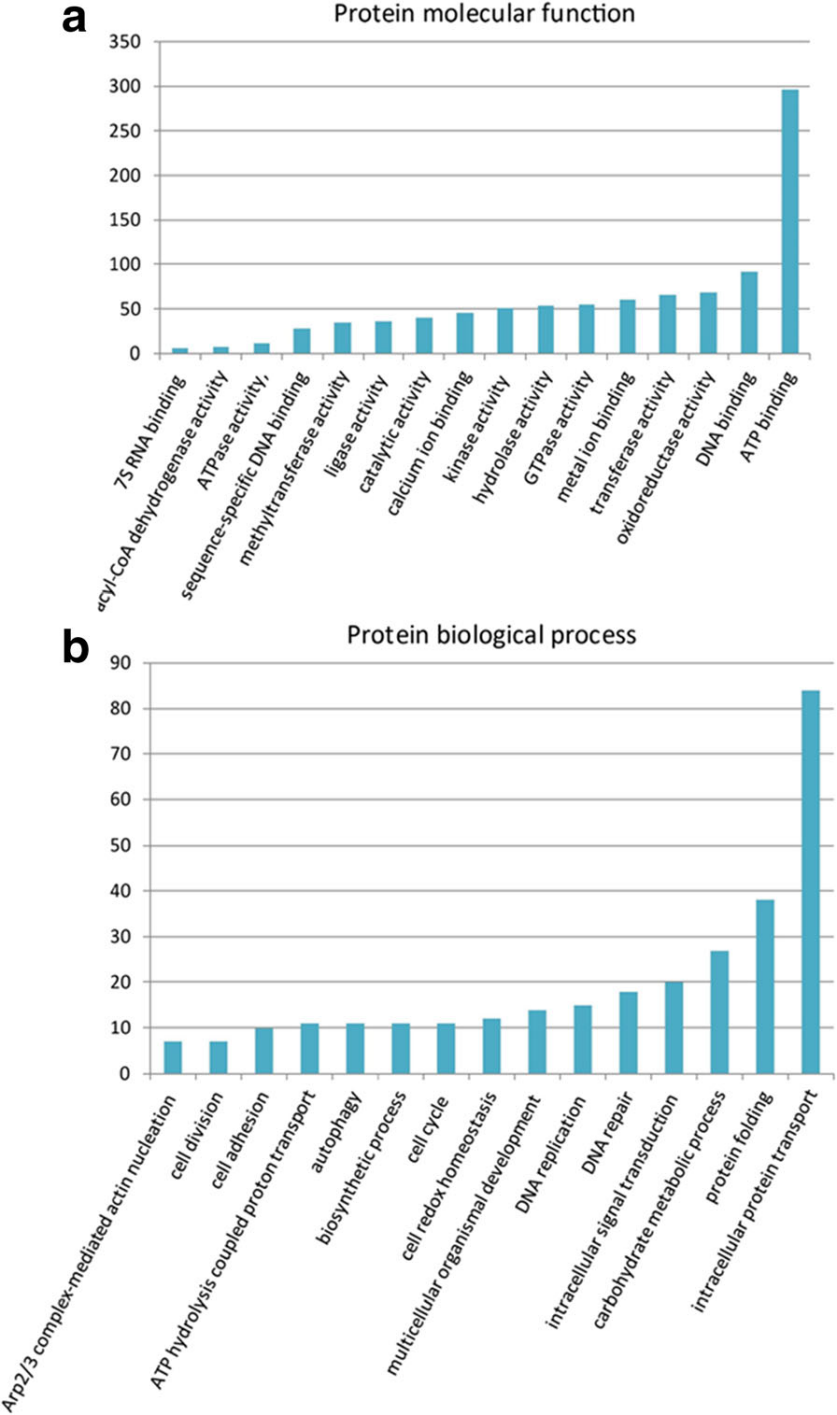
Distribution of molecular functions and biological processes in *S. mimosarum*. **a** Distribution of molecular functions in *S. mimosarum*. **b** Distribution of biological processes in *S. mimosarum*

To understand the biological significances of conserved proteins in the predicted PPI network, genes within the PPI network were annotated using the Gene Ontology (GO) and UniProt online tools. Based on GO classification, 1830 genes were assigned to “biological process”, and 1344 genes were assigned to “molecular function”. Analysis for enriched processes indicated that proteins involved in intracellular protein transport (GO:0006886), protein folding (GO:0006457), and carbohydrate metabolic processes (GO:0005975) are over-represented, likely due to the conserved nature of these processes. In addition, protein folding was enriched due to the protein physical structure requirements for inclusion in the interactome. With the cellular and metabolic processes, a majority of over-represented proteins were involved in carbohydrate metabolism, likely due to the complexity and conservation of this process, along with its being highly studied. In addition, “molecular function” related to ATP binding activity (GO:0005524), DNA binding activity (GO:0003677), oxidoreductase activity (GO:0016491), and transferase activity (GO:0016740) were also over-represented in the PPI network of *S. mimosarum* (Fig. 7). This could also be explained by the high degree of conservation of these functions. Other proteins identified in the PPIs were also involved in significant functions and processes, such as DNA repair, DNA replication, and development of multicellular organisms. A subset of proteins of *S. mimosarum* and their GO annotations were obtained in our analysis; they are provided in Additional files 3 and 4.

### Evolutionary conservation analysis of the PPI sub-networks implicated in RNA splicing across *D. melanogaster*, *H. sapiens*, *A. thaliana*, *S. cerevisiae*, and *S. mimosarum*

We undertook a pathway-based approach to identifying interactions in RNA splicing that are conserved between *D. melanogaster* and *H. sapiens* and between *A. thaliana* and *S. cerevisiae*, which are useful models for studying RNA splicing. Specifically, 42, 19, and 22 RNA splicing-related proteins showed overlap in the PPI networks between *D. melanogaster* and *H. sapiens*, between *D. melanogaster* and *S. cerevisiae*, and between *D. melanogaster* and *A. thaliana*, respectively (Additional file 5). Although RNA splicing processes and related genes perform essential functions in all organisms, the organisms with distant evolutionary relationships showed only partial conservation of RNA splicing PPI processes in *H. sapiens*, *D. melanogaster*, *S. cerevisiae*, and *A. thaliana*. In addition, four overlapping proteins, namely CBP20 (Q9V3L6), LSm7 (Q9VJI7), CBP80 (Q7K4N3), and RE43665p (Q9W2P5), were conserved across *D. melanogaster*, *H. sapiens*, *A. thaliana*, and *S. cerevisiae* [38–40]. Two proteins, Hrp36 (P48810), and SC35 (Q7KTD2), are also regulatory proteins associated with RNA splicing in *D. melanogaster* [41–43]. Hrp36 mainly prevents serine/arginine-rich proteins from promoting the ectopic inclusion of multiple exon variants, which is involved in alternative splicing of the *Dscam* gene [41, 43, 44]. Although Hrp36 has important functions associated with RNA splicing in *D. melanogaster*, it has not been described in *H. sapiens*, *A. thaliana*, or *S. cerevisiae*. In contrast, SC35 is evolutionarily conserved among *D. melanogaster*, *H. sapiens*, and *A. thaliana*. Although a recent study indicated a role of SC35 in alternative mRNA splicing in *D. melanogaster* [42], knowledge regarding the biological functions of this protein is limited.

**Fig. 8:**
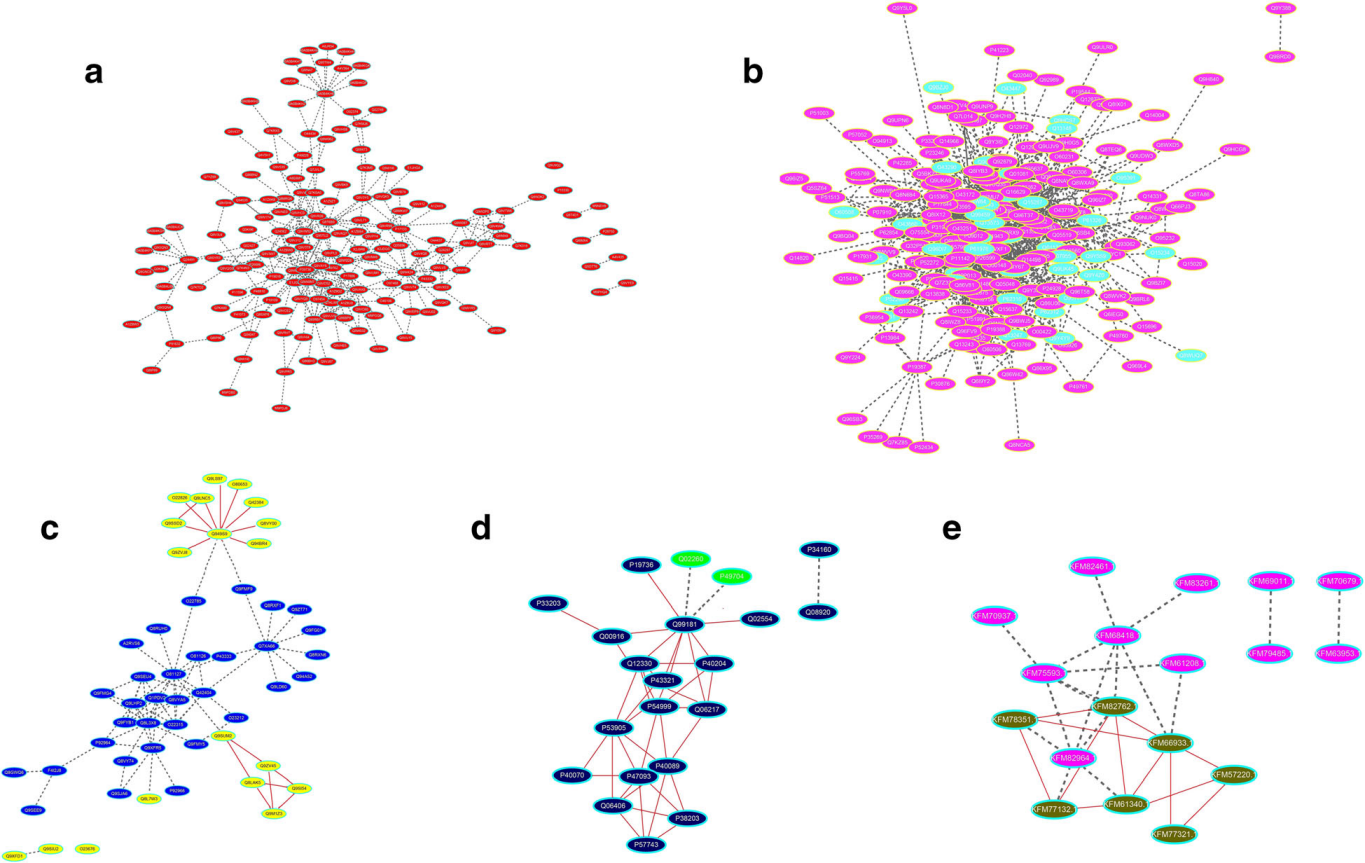
Network protein interactions associated with mRNA splicing in *S. cerevisiae*, *D. melanogaster*, *C*. *jejuni*, *A. thaliana*, and *S. mimosarum*. PPIs associated with RNA splicing were evolutionarily conserved in *D. melanogaster–H. sapiens*, *D. melanogaster–A. thaliana*, and *D. melanogaster–S. cerevisiae* comparisons. **a** Protein interaction network associated with RNA splicing in *D. melanogaster*. *Red* represents interaction network associated with mRNA splicing in *D. melanogaster*. **b** Protein interaction network associated with mRNA splicing in *H. sapiens*. *Pink* represents the non-conservative protein interaction network of RNA splicing in *D. melanogaster*. Cyan represents the conservative protein interaction network associated with RNA splicing in *D. melanogaster*. **c** Network of protein interactions associated with mRNA splicing in *A. thaliana*. *Blue* represents non-conservative protein interaction network associated with RNA splicing in *D. melanogaster*. *Yellow* represents conservative protein interaction network associated with RNA splicing in *D. melanogaster*, while conservative interactions (edges) have been highlighted with *red lines*. **d** Network of protein interactions associated with mRNA splicing in *S. cerevisiae*. *Dark green* represents the conservative protein interaction network associated with RNA splicing in *D. melanogaster*. *Light green* represents the non-conservative protein interaction network associated with RNA splicing in *D. melanogaster*, while conservative interactions (edges) have been highlighted with *red lines*. **e** Predicted network of protein interactions associated with mRNA splicing in *S. mimosarum*. *Green* PPIs in *S. mimosarum* were obtained by prediction based on *S. cerevisiae*, *D. melanogaster*, *H. sapiens*, and *A. thaliana*. Conservative interactions (edges) have been highlighted with *red lines*. *Purple* PPIs in *S. mimosarum* were obtained by prediction based on other model organisms

After analysing the evolutionary conservation of RNA splicing processes in *H. sapiens*, *D. melanogaster*, *S. cerevisiae*, and *A. thaliana*, predicted PPIs associated with RNA splicing in *S. mimosarum* were also analysed in these four model organisms. The overlap in organisms is an indicator of interactions that are likely to occur in *S. mimosarum*; this included three overlapping PPIs in *H. sapiens*, three overlapping PPIs in *D. melanogaster*, a single overlapping PPI in *A. thaliana*, three overlapping PPIs in *S. cerevisiae*, and only one interaction of KFM77132.1 and KFM61340.1 conserved across *D. melanogaster*, *H. sapiens*, and *S. cerevisiae* (Fig. 8; Table 4) [45, 46]. We reanalyzed the data from sub-networks associated with RNA splicing in *S. mimosarum* and found that pre-mRNA-processing-splicing factor 8 (KFM66933.1), which is an important highly connected hub, was also involved in RNA splicing in *S. mimosarum*. This highly connected hub involved in RNA splicing could also be explained by the likely conservation of this pathway in different species and its interactions with other signaling pathways.

**Table 4:** Overlaps of PPIs across *S. mimosarum* and model organisms

*H. sapiens* *S. mimosarum*	*D. melanogaster* *S. mimosarum*	*A. thaliana* *S. mimosarum*	*S. cerevisiae* *S. mimosarum*	*D. melanogaster* *H. sapiens* *S. cerevisiae*
KFM77132.1—KFM61340.1	KFM77132.1--KFM61340.1	KFM57220.1—KFM61340.1	KFM77132.1--KFM61340.1	KFM77132.1--KFM61340.1
KFM66933.1—KFM77321.1	KFM82762.1—KFM77132.1	____	KFM57220.1-- KFM77132.1	____
KFM66933.1—KFM78351.1	KFM57220.1—KFM61340.1	____	KFM57220.1-- KFM61340.1	____

## Discussion

### Evolutionary insights from the predicted PPI network of *S. mimosarum*

*S. mimosarum* belongs to the class Arachnida in the phylum Arthropoda. The *Stegodyphus* species, *S. lineatus*, is estimated to have split from the common ancestor of *S. tentoriicola* and *S. mimosarum* 21 million years ago, and the split between *S. mimosarum* and *S. tentoriicola* is estimated to have occurred 15 million years ago. To gain insight into species’ origins, although the number of PPIs identified in the analysis of individual species is low, it is possible to predict interactions in other species with greater confidence by considering PPIs that overlap in multiple species into consideration. The predicted PPIs in *S. mimosarum* depend on the timing of the split from a common ancestor, and mapping in different organisms is a major research approach to the evolution of all arthropod PPIs.

Our analysis in *S. mimosarum* confirmed 58,489 predicted PPIs in a connected network, and predicted PPIs were systematically examined using overlapping orthologue pairs. In the present study, the results indicated the tendency for a large number of genome-wide proteins in *S. mimosarum* to be mapped to orthologs in organisms and thus identified large numbers of PPIs. These results depend on the consistency of substantial data regarding orthologous pairs with corresponding PPI data in *S. mimosarum*.

### Topology and features of the predicted interactome of *S. mimosarum*

The PPI network in *S. mimosarum* was proposed based on predicted PPIs in this species. *S. mimosarum* was shown to possess a complex PPI network with many highly connected hubs. It is possible that conserved orthologues of these highly connected hubs participate in similar PPIs in other species. Most of the top 20 highly connected nodes of *S. mimosarum* overlapped with four or five of the organisms *D. melanogaster*, *M. musculus*, *A. thaliana*, *S. cerevisiae*, *C. elegans*, *H. sapiens*, *E. coli*, and *C*. *jejuni*. Although these nodes were associated with relatively few functions according to the GO classification, the most highly represented in the interactome were related to DNA binding, structural constituents of the ribosome, ubiquitin–proteasome system, and ATP binding. To analyze the topology, the hub types were computed for the PPI networks in *S. cerevisiae*, *M. musculus*, *P. machaon*, *C*. *jejuni*, *C. elegans*, *D. melanogaster*, and *S. mimosarum*, ranging from hubs with free ends to super-hubs. Our analyses indicated that the tendency toward medium-connection hubs in *S. mimosarum* was similar to those of PPIs in *S. cerevisiae* and *C*. *jejuni*, in which the network model classification depends mainly on the large hubs and hub distribution density. In earlier studies, the “party and date” hub model was commonly adopted for interaction networks. Interaction partner co-expression patterns can distinguish “date hubs” from “party hubs”. The most important role of “date hubs” is the integration of dense sub-networks into a global network topology [47, 48]. However, another view of networks is more akin to the “stratus cloud” model [49, 50], which is supported by the network topology in *S. cerevisiae* [51–55]. Because hub type distribution in *S. mimosarum* was similar to those of PPIs in *S. cerevisiae*, we suggest that the network in *S. mimosarum* might have a greater resemblance to the “stratus cloud” model. This also suggests that proteins have multiple functions or are associated with multiple complexes in the whole network and that signaling pathways overlap or share sub-cycles.

### Analysis of PPI in the RNA splicing process across model organisms and *S. mimosarum*

RNA splicing networks are also conserved in different species, despite large evolutionary distances between them. The predicted PPIs in *S. mimosarum* were obtained from conserved networks without any experimental data using gene mapping techniques. Some conserved proteins of PPI networks involved in RNA splicing participate in this process across *H. sapiens*, *D. melanogaster*, *A. thaliana*, and *S. cerevisiae*, e.g., CBP20, LSm7, CBP80, and RE43665p. However, these four conserved proteins have not been reported to be related to functions in primary regulation of alternative splicing. Hrp36 and SC35 are two important regulatory proteins in RNA splicing in *D. melanogaster* [43, 56–59]. However, Hrp36 and SC35 are not conserved proteins involved in RNA splicing among *H. sapiens*, *A. thaliana*, and *S. cerevisiae*. One possible reason is that the important proteins associated with alternative splicing are different in *D. melanogaster*, *H. sapiens*, *A. thaliana*, and *S. cerevisiae*. Additionally, specific sets of proteins might be associated with alternative splicing in these organisms. Taken together, we preliminarily integrated a pathway associated with RNA splicing using this method. In future studies, difficult problems associated with RNA splicing from other organisms might be resolved based on predictions with abundant PPI resources.

## Conclusions

We predicted 3810 interactome components in *S. mimosarum* using model organism PPI databases, with the numbers of *S. mimosarum* interactome components determined from the numbers of model organism PPIs and *S. mimosarum* orthologues. The “stratus cloud” topology model and small-world properties were analysed in *S. mimosarum* based on the topology model in *S. cerevisiae*. The RNA splicing PPI sub-network showed evolutionary conservation across *H. sapiens*, *D. melanogaster*, *A. thaliana*, and *S. cerevisiae*. In addition, sub-networks associated with RNA splicing in *S. mimosarum* were mainly predicted from *H. sapiens*, *S. cerevisiae*, *A. thaliana*, and *D. melanogaster*. Although model organism PPI networks associated with RNA splicing provided a rich resource, the number of predicted PPIs associated with RNA splicing in *S. mimosarum* is still low. In conclusion, the predicted PPI network of *S. mimosarum* expands the possibility of comparative analyses with other species, thus providing additional insight into network evolution among species.

## Additional files

Additional file 1: Predicted PPI proteins in *S. mimosarum.* Sheet 1: Predicted PPI proteins in *S. mimosarum*; Sheet 2: Predicted PPIs in *S. mimosarum*; Sheet 3: Predicted PPIs of *S. mimosarum* from PPI orthologs in *D. melanogaster*; Sheet 4: Predicted PPIs of *S. mimosarum* from PPI orthologs in *H. sapiens*; Sheet 5: Predicted PPIs of *S. mimosarum* from PPI orthologs in *S. cerevisiae. (XLSX 5485 kb)*

Additional file 2: Database of top 20 nodes. (XLSX 11 kb)

Additional file 3: GO molecular function annotation in *S. mimosarum*. (XLSX 128 kb)

Additional file 4: GO biological process annotation in *S. mimosarum*. (XLSX 93 kb)

Additional file 5: List of conserved genes associated with RNA-splicing PPI across *D. melanogaster*, *H. sapiens*, *A. thaliana*, and *S. cerevisiae* comparisons. (XLSX 13 kb)

## Abbreviations

HSP: Heat-shock protein
PPI: Protein-protein interaction
